# Trajectory of absolute neutrophil counts in patients treated with pegfilgrastim on the day of chemotherapy versus the day after chemotherapy

**DOI:** 10.1007/s00280-016-2970-5

**Published:** 2016-02-17

**Authors:** Yanli Li, Zandra Klippel, Xiaolong Shih, Hong Wang, Maureen Reiner, John H. Page

**Affiliations:** Center for Observational Research, Amgen Inc., 1150 Veterans Blvd, South San Francisco, CA 94080 USA; Clinical Development, Amgen Inc., Thousand Oaks, CA USA; SimulStat Inc., San Diego, CA USA; TechData Service Company, LLC, King of Prussia, PA USA; Global Biostatistical Science, Amgen Inc., Thousand Oaks, CA USA; Center for Observational Research, Amgen Inc., Thousand Oaks, CA USA

**Keywords:** Area over the curve, Chemotherapy-induced neutropenia, Febrile neutropenia, Next-day administration, Pegfilgrastim, Same-day administration

## Abstract

**Purpose:**

Risk of infection increases with severity and duration of chemotherapy-induced neutropenia (CIN). Pegfilgrastim is approved for use on the day after chemotherapy to reduce incidence of infection, as manifested by febrile neutropenia (FN), in patients receiving myelosuppressive chemotherapy. In this study, we compared severity and duration of absolute neutrophil count (ANC) suppression in patients who received pegfilgrastim on the same day as chemotherapy versus the next day.

**Methods:**

We combined individual patient data from four Amgen-sponsored clinical trials in which patients with cancer were randomized to receive pegfilgrastim either the same day as chemotherapy or the next day. Severity and duration of ANC suppression were calculated using area over the curve (AOC, the area over the ANC–time response curve and below a given clinical threshold). AOC of ANC and incidences of CIN and FN were compared by day of pegfilgrastim use.

**Results:**

The analysis included 95 same-day patients and 97 next-day patients. Despite similar ANC at baseline, ANC at nadir was higher among next-day patients than same-day patients. Mean AOC of ANC (cutoff 0.5 × 10^9^/L) among next-day patients was lower by 0.30 (95 % confidence interval: 0.16, 0.43) 10^9^/L × day than same-day patients in cycle 1. Next-day patients had lower incidences of CIN than same-day patients, but there were no significant differences in incidences of FN.

**Conclusions:**

Patients who received pegfilgrastim the day after chemotherapy had less severe and shorter suppression of ANC than patients who received pegfilgrastim the same day as chemotherapy.

**Electronic supplementary material:**

The online version of this article (doi:10.1007/s00280-016-2970-5) contains supplementary material, which is available to authorized users.

## Introduction

Neutrophils, the most abundant leukocytes in circulation, play a crucial role in innate immune responses against infections [1]. Cytotoxic chemotherapy suppresses the hematopoietic system and may lead to chemotherapy-induced neutropenia (CIN), a condition that makes patients vulnerable to potentially life-threatening infections [2]. Following initiation of myelosuppressive chemotherapy, absolute neutrophil count (ANC) follows a trajectory that includes a decline to its lowest point (the nadir) and subsequent rise as the bone marrow recovers [3]. Lower ANC (or leukocytes) at nadir and longer duration of severe CIN (or leukopenia) have been shown to be associated with higher risk of infection [4–6].

Neutropenia blunts the inflammatory response to nascent infections and reduces the signs and symptoms of infection; therefore, the only sign of infection in the presence of neutropenia is often fever [2]. Febrile neutropenia (FN), the combination of neutropenia and fever, is a serious toxicity of myelosuppressive chemotherapy that can lead to chemotherapy dose delays and reductions as well as increased morbidity, mortality, and healthcare resource use [7–9].

Granulocyte colony-stimulating factor (G-CSF) regulates the production of neutrophils within the bone marrow and induces proliferation and differentiation of neutrophil precursors [10, 11]. Pegfilgrastim (Neulasta^®^, Amgen Inc., Thousand Oaks, CA, USA) is a pegylated recombinant human G-CSF that is indicated to decrease the incidence of infection, as manifested by FN, in patients with non-myeloid malignancies receiving myelosuppressive anti-cancer drugs [12, 13].

Neulasta^®^ prescribing information specifies that pegfilgrastim should not be administered between 14 days before and 24 h after administration of chemotherapy [13]. Theoretically, the simultaneous administration of exogenous G-CSF and chemotherapy may lead to an increased pool of neutrophil precursors susceptible to destruction by chemotherapy, leading paradoxically to an increased risk of neutropenia [14, 15]. Nevertheless, some patients still receive pegfilgrastim on the same day as chemotherapy rather than the next day [16–18].

In the current study, we pooled individual patient data from four Amgen-sponsored clinical trials in which patients were randomized to receive pegfilgrastim on the same day as chemotherapy versus the next day. The objectives of this study were to compare several metrics for severity and duration of ANC suppression and incidence proportions of CIN and FN among patients who received pegfilgrastim on the same day as chemotherapy versus the next day.

## Materials and methods

### Study design and data source

The current study is a secondary analysis of individual patient data collected in four randomized phase 2 clinical trials sponsored by Amgen Inc. The trials were conducted between 2003 and 2005 in patients with non-Hodgkin’s lymphoma, breast cancer, relapsed or refractory ovarian cancer, and advanced or metastatic non-small cell lung cancer. The primary objective of the trials was to provide data on the safety and efficacy of pegfilgrastim administered on the same day as chemotherapy (within 24 h of chemotherapy completion) versus the next day (24 h after chemotherapy completion). The primary efficacy endpoint of all four trials was duration of grade 4 neutropenia. Criteria for the inclusion of these four trials in this analysis are shown in Supplementary material 1. Key information regarding these four trials is summarized in Supplementary material 2. In three of four trials (Amgen studies 20020134, 20020778, and 20030123), chemotherapy was administered only on day 1 of the chemotherapy cycle, and the same-day and next-day patients received pegfilgrastim on day 1 and day 2 of the cycle, respectively. In Amgen study 20030122, chemotherapy was administered over the first 5 days of the chemotherapy cycle, and the same-day and next-day patients received pegfilgrastim on day 5 and day 6 of the cycle, respectively (Supplementary material 2).

### Study population

Patients were included in this analysis if they were enrolled in one of the four aforementioned randomized clinical trials and met all of the following additional inclusion criteria: baseline ANC ≥ 1500/µL at initiation of chemotherapy, ANC measured at least four times per cycle in at least one cycle of the chemotherapy course under study, and normal body temperature (<38 °C) at initiation of chemotherapy. Patients were excluded if they had an active infection that required treatment with anti-infectives within 72 h of chemotherapy, received prophylactic antibiotics, received pelvic irradiation or radiation therapy extending beyond a single involved field within 4 weeks of chemotherapy initiation, or had a prior malignancy in the previous 5 years.

Patients in two of the included clinical trials (Amgen studies 20020778 and 20030122) had ANC measured at least four times per cycle in both cycle 1 and cycle 3. Patients in the other two trials (Amgen studies 20020134 and 20030123) had ANC measured at least four times per cycle only in cycle 1. All the ANC-related analyses in the current study were conducted in cycles in which ANC was measured at least four times per cycle.

### Endpoints

**Fig. 1:**
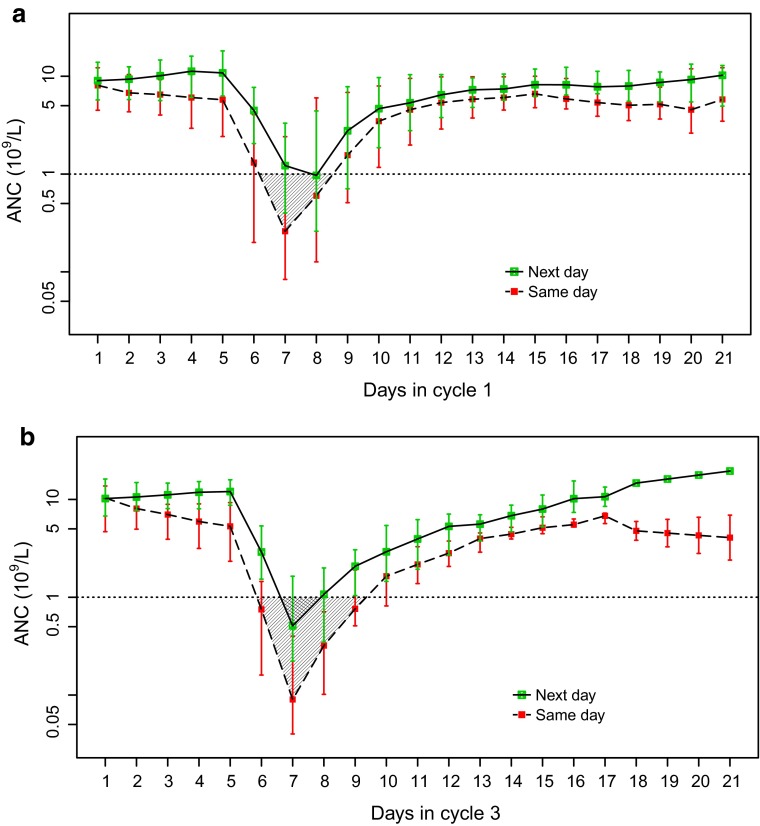
ANC trajectory in patients who received pegfilgrastim on the same day as chemotherapy versus the next day. **a** ANC trajectory in cycle 1. Data are from eligible patients in Amgen studies 20020134, 20020778, 20030122, and 20030123. **b** ANC trajectory in cycle 3. Data are from eligible patients in Amgen studies 20020778 and 20030122. *Red squares* represent daily median ANC values for patients who received pegfilgrastim on the same day as chemotherapy. *Green squares* represent daily median ANC values for patients who received pegfilgrastim on the day after chemotherapy. *Error bars* represent Q1 and Q3 of daily ANC values. *AOC* is the area above the ANC-time response curve and below a given clinical threshold (ANC < 1.0 × 10^9^/L or ANC < 0.5 × 10^9^/L). The *cross-hatched area* represents AOC for ANC < 1.0 × 10^9^/L. ANC values are shown on a natural logarithmic scale. Days are numbered from chemotherapy initiation: day 1 is the day of chemotherapy; day 2 is the day after chemotherapy. *ANC* absolute neutrophil count; *AOC* area over the *curve*; *Q1* quartile 1; *Q3* quartile 3

The primary endpoint of this study was area over the ANC–time response curve (AOC). AOC of ANC is the area above the ANC–time response curve and below the thresholds of 0.5 × 10^9^/L or 1.0 × 10^9^/L in a given chemotherapy cycle (shown graphically in Fig. 1). AOC of ANC measures both severity and duration of neutropenia. The more severe and the longer the duration of neutropenia, the higher the AOC. The thresholds of 0.5 × 10^9^/L and 1.0 × 10^9^/L are based on the Common Terminology Criteria for Adverse Events (CTCAE): an ANC < 0.5 × 10^9^/L is categorized as grade 4 neutropenia, while an ANC between 0.5 × 10^9^/L and 1.0 × 10^9^/L is categorized as grade 3 neutropenia [19].

The secondary endpoints of this study were ANC at nadir, time to ANC nadir, and incidence proportions of grade 4 CIN (ANC < 0.5 × 10^9^/L), grade 3/4 CIN (ANC < 1.0 × 10^9^/L), grade 4 FN, and grade 3/4 FN within a chemotherapy cycle. Grade 4 and grade 3/4 FN were defined as an infectious episode (body temperature ≥38.2 °C, infection-related hospitalization, or infection-related adverse event) occurring on the same day or within 1 day of grade 4 or grade 3/4 neutropenia, respectively.

### Statistical analysis

Descriptive analyses were conducted to characterize demographics, disease characteristics, and chemotherapy treatments in the overall study population and in each of the treatment groups (same-day versus next-day pegfilgrastim use). The two-sample *t* test was used to assess differences in continuous variables, and the Chi-square test was used to assess differences in categorical variables between the treatment groups. No multiplicity adjustment was used, and *p* values should be considered nominal. Body surface area was calculated using the Mosteller formula [20]. Risk of FN for each chemotherapy regimen was based on the National Comprehensive Cancer Network (NCCN) guidelines [21]. For regimens that remain unclassified, FN incidence in the placebo arms (no G-CSF) of Amgen-sponsored clinical trials and FN risk reported in the literature were used to determine FN risk category [22].

Baseline ANC values, ANC at nadir, and time to ANC nadir were described by treatment group for each cycle with ≥4 ANC measurements (cycles 1 and 3). The log-linear interpolation technique [23] was used to derive ANC on days without a measurement using the adjacent ANC measurements from each patient. ANC nadir was the lowest ANC value that occurred during the chemotherapy cycle. Time to ANC nadir was calculated as the number of days from chemotherapy initiation to ANC nadir. Distributions of all ANC metrics were skewed; thus, the Wilcoxon rank sum test was used to compare the differences between the two treatment groups. Differences in the distribution of time to ANC nadir (<7, 7, 8, and >8 days) between the two groups were tested using the Chi-square test or Fisher’s exact test (if expected cell frequency was <5). A sensitivity analysis was performed to compare time to ANC nadir for the studies in which chemotherapy was administered only on day 1 of the chemotherapy cycle (Amgen studies 20020134, 20020778, and 20030123), and a separate sensitivity analysis was performed to compare time to ANC nadir for the study in which chemotherapy was administered over the first 5 days of the chemotherapy cycle (Amgen study 20030122).

AOC of the ANC–time response curve (below the thresholds of 0.5 × 10^9^/L or 1.0 × 10^9^/L) was calculated using the Riemann sum method, assuming ANC values to be constant within each day [24]. Due to the large proportion of patients with an AOC of 0 (e.g., 35.8 % of same-day patients and 51.0 % of next-day patients had an AOC of 0 below the threshold of 0.5 × 10^9^/L in cycle 1), median AOC might not be meaningful. Mean AOC of ANC was therefore calculated for each group as were differences in mean AOC of ANC between patients who received pegfilgrastim on the same day as chemotherapy versus the next day. Differences in mean AOC were calculated in cycle 1 or cycle 3 separately using linear regression and in the two cycles combined using linear mixed-effect regression to control for within-patient and within-study correlations. Age (as a linear continuous variable) and Eastern Cooperative Oncology Group (ECOG) performance status (0, 1, and 2–3 as a categorical variable), the covariates with imbalanced distributions between the two groups, were controlled for in the adjusted model. A bootstrap procedure was used to estimate the 95 % confidence intervals (CIs) for mean AOC difference. One thousand bootstrap samples were first selected from AOC estimates using stratified random sampling (by day of pegfilgrastim use and study) with replacement. Mean AOC differences by day of pegfilgrastim use within each of the 1000 replicates were then estimated. The 2.5th and 97.5th percentiles of the bootstrap samples were used as the 95 % CIs of the mean AOC difference.

Incidence proportions of CIN and FN within a chemotherapy cycle were calculated by day of pegfilgrastim use for cycles 1 and 3. A generalized linear mixed model with logit link function was used to compare the odds of CIN and FN in next-day versus same-day patients, adjusting for age (as a linear continuous variable) and ECOG performance status (0, 1, and 2–3 as a categorical variable) in cycle 1, cycle 3, and cycles 1 and 3 combined. Within-study correlation and within-patient correlation in the combined analysis of cycles 1 and 3 were controlled for by including random intercepts in the mixed model.

## Results

### Clinical trials and patients

Four randomized phase 2 clinical trials were identified in which patients were allocated to receive pegfilgrastim on the same day as chemotherapy versus the next day. A total of 192 patients in these trials were eligible for inclusion in the current study: 95 patients who received pegfilgrastim on the same day as chemotherapy and 97 who received pegfilgrastim on the next day. Patient disposition by inclusion and exclusion criteria for each of the studies and for all studies combined is shown in Table 1.

**Table 1:** Patient disposition after applying each of the inclusion/exclusion criteria

Inclusion/exclusion criteria	Study 20020134 NHL		Study 20020778 Breast Cancer		Study 20030122 Ovarian Cancer		Study 20030123 NSCLC		All studies	
	Same day	Next day	Same day	Next day	Same day	Next day	Same day	Next day	Same day	Next day
Patients enrolled and received assigned drugs	36	39	45	43	8	11	43	44	132	137
Baseline ANC ≥ 1500/µL	36	37	45	43	8	11	43	44	132	135
Normal baseline body temperature	35	37	45	43	8	11	43	44	131	135
No history of cancer within the last 5 years	30	30	32	32	6	7	38	34	106	103
No history of chemotherapy	30	30	32	32	5	7	38	34	105	103
No recent radiation therapy	30	30	32	31	5	7	34	33	101	101
ANC measured ≥4 times in cycle 1	29	29	32	30	5	6	29	32	95	97

All numbers indicate number of patients

*ANC* absolute neutrophil count, *NHL* non-Hodgkin’s lymphoma; *NSCLC* non-small cell lung cancer

Of the eligible patients, most were female (67.7 %), white (80.7 %), and had an ECOG performance status of 0 (54.7 %). Mean (±standard deviation [SD]) age of the patients was 58.9 (±12.7) years. Primary tumor types were breast cancer (32.3 %), non-small cell lung cancer (31.8 %), non-Hodgkin’s lymphoma (30.2 %), and ovarian cancer (5.7 %). About half of the patients (48.4 %) had advanced tumors (stage IV or “extensive”). Most patients (62.0 %) received chemotherapy regimens with an intermediate risk (10–20 %) of FN. Demographics, disease characteristics, and chemotherapy regimen FN risk are shown in Table 2. Overall, demographics and disease characteristics were balanced between the same-day and next-day groups.

**Table 2:** Baseline demographics, disease characteristics, and treatment parameters of the study population

	Overall (*N* = 192)	Same day (*N* = 95)	Next day (*N* = 97)	*p* value^a^
Sex, *n* (%)				
Male	62 (32.3)	31 (32.6)	31 (32.0)	0.921
Female	130 (67.7)	64 (67.4)	66 (68.0)	
Race, *n* (%)				
White or Caucasian	155 (80.7)	77 (81.1)	78 (80.4)	0.180
Black or African American	21 (10.9)	13 (13.7)	8 (8.2)	
Other	16 (8.3)	5 (5.3)	11 (11.3)	
Age, years				
Mean ± SD	58.9 ± 12.7	57.5 ± 12.7	60.4 ± 12.7	0.114
ECOG performance status, *n* (%)				
0	105 (54.7)	58 (61.1)	47 (48.5)	0.191
1	82 (42.7)	35 (36.8)	47 (48.5)	
2	5 (2.6)	2 (2.1)	3 (3.1)	
BSA, m^2^				
Mean ± SD	1.86 ± 0.24	1.87 ± 0.24	1.86 ± 0.25	0.739
BMI, kg/m^2^				
Mean ± SD	27.34 ± 5.76	27.07 ± 5.57	27.61 ± 5.95	0.522
Primary tumor type, *n* (%)				
Breast cancer	62 (32.3)	32 (33.7)	30 (30.9)	0.963
Non-small cell lung cancer	61 (31.8)	29 (30.5)	32 (33.0)	
Non-Hodgkin’s lymphoma	58 (30.2)	29 (30.5)	29 (29.9)	
Ovarian cancer	11 (5.7)	5 (5.3)	6 (6.2)	
Tumor stage^b^, *n* (%)				
Non-advanced	99 (51.6)	51 (53.7)	48 (49.5)	0.560
Advanced	93 (48.4)	44 (46.3)	49 (50.5)	
Chemotherapy regimen, *n* (%)				
Intermediate risk of FN^c^	119 (62.0)	58 (61.1)	61 (62.9)	0.794
High risk of FN^d^	73 (38.0)	37 (38.9)	36 (37.1)	

*BMI* body mass index, *BSA* body surface area, *ECOG* eastern cooperative oncology group, *FN* febrile neutropenia, *SD* standard deviation

^a^Two-sample *t* test was used to test differences for continuous variables, and Chi-square test or Fisher’s exact test (if expected cell frequency was <5) were used to test differences for categorical variables between same-day and next-day patients. No multiplicity adjustment was used and *p* values should be considered nominal

^b^Stages I, II, and III or “limited” were classified as non-advanced; stage IV or “extensive” were classified as advanced

^c^Regimens with an intermediate risk (10–20 %) of FN included: 21-day R-CHOP (cyclophosphamide, doxorubicin, vincristine, prednisone, and rituximab) and 21-day carboplatin and docetaxel

^d^Regimens with a high risk (>20 %) of FN included: 21-day TAC (docetaxel, doxorubicin, and cyclophosphamide) and 21-day topotecan

### ANC trajectory

ANC trajectories of the patients who received pegfilgrastim on the same day as chemotherapy and those who received pegfilgrastim the next day are shown in Fig. 1. In both cycle 1 (Fig. 1a) and cycle 3 (Fig. 1b), the ANC trajectories of same-day patients and next-day patients began to diverge on the day after chemotherapy (day 2). ANC at nadir was lower among same-day patients than among next-day patients in both cycle 1 and cycle 3. ANC values returned to baseline sooner and remained higher throughout the cycle among next-day patients than among same-day patients in both cycles.

Key statistics of the ANC trajectory of patients in this study are shown in Table 3. Baseline ANC values were not different between same-day and next-day patients (*p* > 0.05). In contrast, ANC at nadir was significantly lower among same-day patients than among next-day patients in both cycle 1 (median [Q1, Q3]: 0.13 [0.04, 1.31] versus 0.54 [0.11, 2.04] × 10^9^/L, *p* = 0.003) and cycle 3 (median [Q1, Q3]: 0.07 [0.04, 0.27] versus 0.37 [0.14, 1.00] × 10^9^/L, *p* < 0.001). Although the mean or median time to ANC nadir was similar between the two treatment groups in cycle 1, same-day patients tended to reach ANC nadir earlier than next-day patients: 22.1 versus 7.3 % reached ANC nadir within 7 days after chemotherapy in cycle 1 (Table 3). No significant differences in time to ANC nadir were observed in cycle 3 (Table 3). In the sensitivity analysis, we observed that same-day patients tended to reach ANC nadir earlier than next-day patients in cycle 1 for studies in which chemotherapy was administered only on day 1 of the chemotherapy cycle (Supplementary material 3). Only one study with a very small sample size (*n* = 11 in cycle 1, *n* = 7 in cycle 3) had chemotherapy administered over multiple days of the chemotherapy cycle. In this study, no difference in time to ANC nadir was observed between same-day and next-day patients (Supplementary material 4).

**Table 3:** ANC trajectory in patients who received pegfilgrastim on the same day as chemotherapy versus the next day in cycle 1 and cycle 3

ANC trajectory metrics	Same day	Next day	*p* value^a^
*Cycle 1*			
Baseline ANC (10^9^/L)			
Mean ± SD (*n* ^b^)	8.99 ± 5.78 (94)	9.64 ± 5.98 (96)	
Median (Q1, Q3)	7.14 (4.42, 12.18)	8.08 (5.31, 13.39)	0.404
ANC at nadir (10^9^/L)			
Mean ± SD (*n* ^b^)	1.33 ± 2.32 (95)	1.83 ± 2.96 (96)	
Median (Q1, Q3)	0.13 (0.04, 1.31)	0.54 (0.11, 2.04)	**0.003**
Time to ANC nadir (days)			
Mean ± SD (*n* ^b^)	7.52 ± 2.48 (95)	7.61 ± 1.43 (96)	
Median (Q1, Q3)	7.00 (7.00, 8.00)	7.00 (7.00, 8.00)	**0.019**
Time to ANC nadir distribution, *n* (%)			
<7 days	21 (22.1)	7 (7.3)	**0.028**
7 days	40 (42.1)	43 (44.8)	
8 days	23 (24.2)	30 (31.3)	
>8 days	11 (11.6)	16 (16.7)	
*Cycle 3*			
Baseline ANC (10^9^/L)			
Mean ± SD (*n* ^b^)	8.65 ± 4.74 (30)	9.32 ± 5.33 (33)	
Median (Q1, Q3)	8.67 (4.92, 12.14)	7.99 (4.34, 13.90)	0.549
ANC at nadir (10^9^/L)			
Mean ± SD (*n* ^b^)	0.27 ± 0.50 (30)	0.74 ± 0.93 (33)	
Median (Q1, Q3)	0.07 (0.04, 0.27)	0.37 (0.14, 1.00)	**<0.001**
Time to ANC nadir (days)			
Mean ± SD (*n* ^b^)	7.53 ± 0.73 (30)	7.70 ± 0.88 (33)	
Median (Q1, Q3)	7.00 (7.00, 8.00)	7.00 (7.00, 8.00)	0.503
Time to ANC nadir distribution^c^ *n* (%)			
≤7 days	18 (60.0)	17 (51.5)	0.829
8 days	8 (26.7)	11 (33.3)	
>8 days	4 (13.3)	5 (15.2)	

Analyses in cycle 1 include data from eligible patients in Amgen studies 20020134, 20020778, 20030122, and 20030123 and in cycle 3 include data from eligible patients in Amgen studies 20020778 and 20030122

Bold indicates *p* < 0.05

*ANC* absolute neutrophil count; *Q1* quartile 1; *Q3* quartile 3; *SD* standard deviation

^a^Wilcoxon rank sum test was used to test differences of baseline ANC, ANC at nadir, and time to nadir (continuous), and Chi-square test or Fisher’s exact test (if expected cell frequency was <5) was used to test difference of time to nadir distribution (<7 days, 7 days, 8 days, and >8 days) between same-day versus next-day patients

^b^The *n* for each parameter (of each treatment arm) is the number of patients eligible for the corresponding statistics in cycle 1 or cycle 3

^c^No patients reached ANC nadir in <7 days in cycle 3, so <7 days and 7 days were combined

### Area over the ANC–time response curve

AOC of ANC, a composite measure of duration and severity of ANC suppression, was significantly higher among same-day patients than among next-day patients (Table 4). In cycle 1, when ANC < 0.5 × 10^9^/L was used as the threshold, mean AOC of ANC was higher by 0.30 (95 % CI 0.16, 0.43) 10^9^/L × day among same-day patients than among next-day patients. When ANC < 1.0 × 10^9^/L was used as the threshold, mean AOC of ANC was higher by 0.73 (95 % CI 0.37, 1.05) 10^9^/L × day among same-day patients. In cycle 3, when both ANC < 0.5 × 10^9^/L and ANC < 1.0 × 10^9^/L were used as the thresholds, AOC was significantly higher among same-day patients than among next-day patients. Similar findings were observed in the analysis of cycles 1 and 3 combined (Table 4).

**Table 4:** Comparison of mean AOC of ANC in patients who received pegfilgrastim on the same day as chemotherapy versus the next day in cycle 1 and cycle 3

AOC threshold	Cycle	Day of pegfilgrastim use	Mean ± SD AOC (*n* ^a^) 10^9^/L × day	Crude Mean AOC Difference (95 % CI) 10^9^/L × day	Adjusted Mean AOC^b^ Difference (95 % CI) 10^9^/L × day
<0.5 × 10^9^/L	Cycle 1	Same day	0.65 ± 0.65 (95)	**0.31** (**0.17**, **0.43**)	**0.30** (**0.16**, **0.43**)
		Next day	0.34 ± 0.49 (96)		
	Cycle 3	Same day	0.74 ± 0.50 (30)	**0.47** (**0.28**, **0.64**)	**0.43** (**0.23**, **0.61**)
		Next day	0.27 ± 0.33 (33)		
	Cycles 1 and 3^c^			**0.33** (**0.20**, **0.45**)	**0.36** (**0.22**, **0.49**)
<1.0 × 10^9^/L	Cycle 1	Same day	1.85 ± 1.71 (95)	**0.75** (**0.41**, **1.04**)	**0.73** (**0.37**, **1.05**)
		Next day	1.10 ± 1.31 (96)		
	Cycle 3	Same day	2.23 ± 1.18 (30)	**1.21** (**0.74**, **1.65**)	**1.11** (**0.63**, **1.55**)
		Next day	1.02 ± 0.96 (33)		
	Cycles 1 and 3^c^			**0.82** (**0.50**, **1.12**)	**0.88** (**0.54**, **1.20**)

Mean AOC difference = mean AOC in same-day patients−mean AOC in next-day patients; analyses in cycle 1 include data from eligible patients in Amgen studies 20020134, 20020778, 2003012, and 20030123 and in cycle 3 include data from eligible patients in Amgen studies 20020778 and 20030122

Bold indicates that 95 % CIs for crude or adjusted mean AOC difference do not include 0

*AOC* area over the curve; *ANC* absolute neutrophil count; *CI* confidence interval; *ECOG* Eastern Cooperative Oncology Group; *SD* standard deviation

^a^The *n* for each parameter (of each treatment arm) is the number of patients eligible for the corresponding statistics in cycle 1 or cycle 3

^b^Covariates included in the adjusted model were age and ECOG performance status

^c^Linear mixed-effect regression model was used to calculate mean AOC difference in cycles 1 and 3 combined. Within-study and within-patient correlations were controlled for in the analysis. Bootstrap procedure was used to derive 95 % CIs

### Chemotherapy-induced neutropenia and febrile neutropenia

Incidence proportions of CIN and FN within the chemotherapy cycle among same-day and next-day patients are shown in Table 5. Incidence proportion of grade 4 CIN was significantly lower among next-day patients than among same-day patients in both cycle 1 (49.0 versus 64.2 %, adjusted odds ratio [OR] [95 % CI] 0.23 [0.09, 0.62]) and cycle 3 (57.6 versus 83.3 %, adjusted OR [95 % CI] 0.19 [0.04, 0.79]). In cycles 1 and 3 combined, next-day patients had significantly lower odds of having grade 4 CIN (adjusted OR [95 % CI] 0.23 [0.10, 0.49]).

**Table 5:** Comparison of incidence proportions of CIN and FN in patients who received pegfilgrastim on the same day as chemotherapy versus the next day in cycles 1 and 3

Neutropenic event	Cycle	Day of pegfilgrastim use	Cases/patients (incidence proportion)	Crude OR (95 % CI)	Adjusted OR (95 % CI)^a^
Grade 3/4 CIN	Cycle 1	Same day	69/95 (72.6 %)	Reference	Reference
		Next day	63/96 (65.6 %)	0.58 (0.22, 1.55)	0.48 (0.17, 1.35)
	Cycle 3	Same day	28/30 (93.3 %)	Reference	Reference
		Next day	24/33 (72.7 %)	**0.12** (**0.01, 0.95**)	0.13 (0.02, 1.02)
	Cycles 1 and 3^b^			**0.41** (**0.17, 0.94**)	**0.36** (**0.15, 0.87**)
Grade 4 CIN	Cycle 1	Same day	61/95 (64.2 %)	Reference	Reference
		Next day	47/96 (49.0 %)	**0.31** (**0.12, 0.77**)	**0.23** (**0.09, 0.62**)
	Cycle 3	Same day	25/30 (83.3 %)	Reference	Reference
		Next day	19/33 (57.6 %)	**0.22** (**0.06, 0.87**)	**0.19** (**0.04, 0.79**)
	Cycles 1 and 3^b^			**0.29** (**0.14, 0.60**)	**0.23** (**0.10, 0.49**)
Grade 3/4 FN	Cycle 1	Same day	16/95 (16.8 %)	Reference	Reference
		Next day	10/96 (10.4 %)	0.58 (0.24, 1.40)	0.59 (0.24, 1.45)
	Cycle 3	Same day	2/30 (6.7 %)	Reference	Reference
		Next day	2/33 (6.1 %)	0.90 (0.11, 7.14)	1.02 (0.12, 8.42)
	Cycles 1 and 3^b^			0.62 (0.28, 1.39)	0.66 (0.29, 1.49)
Grade 4 FN	Cycle 1	Same day	16/95 (16.8 %)	Reference	Reference
		Next day	9/96 (9.4 %)	0.51 (0.21, 1.27)	0.53 (0.21, 1.32)
	Cycle 3	Same day	2/30 (6.7 %)	Reference	Reference
		Next day	2/33 (6.1 %)	0.90 (0.11, 7.14)	1.02 (0.12, 8.42)
	Cycles 1 and 3^b^			0.56 (0.25, 1.28)	0.60 (0.26, 1.38)

Analyses in cycle 1 include data from eligible patients in Amgen studies 20020134, 20020778, 20030122, and 20030123 and in cycle 3 include data from eligible patients in Amgen studies 20020778 and 20030122

Bold indicates that 95 % CIs for crude OR or adjusted OR do not include 1

*CI* confidence interval; *CIN* chemotherapy-induced neutropenia; *ECOG* Eastern Cooperative Oncology Group; *FN* febrile neutropenia; *OR* odds ratio

^a^Covariates adjusted in the generalized linear mixed model included age and ECOG performance status

^b^Generalized linear mixed model using logit link function (with random intercepts to control for the within-study and within-patient correlations) was used to calculate the ORs (95 % CIs) for cycles 1 and 3 combined

Incidence proportion of grade 3/4 CIN was not significantly different between same-day versus next-day patients in cycles 1 and 3 separately: cycle 1 (65.6 versus 72.6 %, adjusted OR [95 % CI] 0.48 [0.17, 1.35]) and cycle 3 (72.7 versus 93.3 %, adjusted OR [95 % CI] 0.13 [0.02, 1.02]). However, in cycles 1 and 3 combined, the incidence proportion of grade 3/4 CIN was statistically lower among next-day patients than among same-day patients (adjusted OR [95 % CI] 0.36 [0.15, 0.87]).

No statistically significant differences were observed between same-day and next-day patients in the incidence proportions of grade 4 FN or grade 3/4 FN in cycle 1, cycle 3, or cycles 1 and 3 combined (Table 5).

## Discussion

Several randomized, placebo-controlled clinical trials have shown that patients with cancer who were treated with chemotherapy and prophylactic G-CSF experienced substantially less severe suppression of ANC, more rapid recovery of ANC, and lower incidence of infection (characterized by FN) than patients who did not receive prophylactic G-CSF [12, 25, 26]. In the current study, patients who received pegfilgrastim prophylaxis on the day after chemotherapy (24 h after chemotherapy completion) had a less severe fall in ANC and more rapid recovery of ANC than patients who received pegfilgrastim on the same day as chemotherapy (within 24 h of chemotherapy completion).

Previous studies have provided some evidence that patients with cancer who had lower ANCs and longer duration of severe CIN following chemotherapy were at higher risk of developing infection [4, 27]. Each unit increase in AOC of ANC (10^9^/L × day) below the threshold of ANC < 0.5 × 10^9^/L was found to be associated with a significantly increased risk of infection-related hospitalization (hazard ratio [95 % CI] 1.98 [1.35, 2.90]) [28]. In this study, we did not find a statistically significant difference in the incidences of FN between patients who received pegfilgrastim prophylaxis on the same day as chemotherapy versus the next day. This is likely due to the limited statistical power of the study. The study included 192 patients and had about 30 % power to detect a relative risk of 0.6.

Burris et al. [29] analyzed ANC data from the same four clinical trials included in this analysis; however, the objectives and analytical approaches of that study were different from the current study. Burris et al. presented ANC data, such as ANC nadir and incidence and duration of grade 4 neutropenia in cycle 1, for each individual trial. We pooled individual patient data from the four trials and performed a statistical analysis of the shape of the ANC trajectories by using AOC of ANC. Two additional clinical trials evaluated the difference between same-day and next-day pegfilgrastim prophylaxis. Saven et al. [30] reported higher incidence of grade 4 CIN among same-day patients but similar incidence of FN, while Belani et al. [31] reported no difference in the incidences of CIN or FN by day of pegfilgrastim use. Results from observational studies are also inconsistent, which might be explained by heterogeneous study designs, possible selection bias, and confounding [18, 32–35]. Also, most of the observational studies had relatively small sample sizes [32–35]. One recent observational study retrospectively analyzed 45,592 patients (4336 same day, 32,759 next day) from two private US healthcare claims databases. The study reported that odds of FN were significantly higher among patients who received pegfilgrastim on the same day as chemotherapy versus the next day (OR [95 % CI] 1.6 [1.3, 1.9] for cycle 1; OR [95 % CI] 1.5 [1.3, 1.6] for all cycles combined) [18]. The direction and magnitude of the associations reported in that study are similar to those reported here.

The 2015 update to the American Society of Clinical Oncology Clinical Recommendations for the Use of White Blood Cell (WBC) Growth Factors states that, “Evidence suggests that pegfilgrastim administered 1–3 days after chemotherapy results in a lower risk of infection than pegfilgrastim administered on the same day as chemotherapy” [36]. The current version of the NCCN Guidelines^®^ for Myeloid Growth Factors states that, “Beginning pegfilgrastim the day after chemotherapy is preferred” [21]. The favorable ANC trajectory and lower incidence proportion of CIN observed in the current study support these recommendations.

This study has several strengths. By performing a pooled analysis of clinical trial data from patients who were randomized to receive pegfilgrastim on the same day as chemotherapy or the next day, we avoided bias due to confounding, an issue that might affect other study designs. Our data included frequent measurements of ANC, which enabled good estimation of ANC trajectory and of the difference in AOC of ANC between the patients who received pegfilgrastim on the same day as chemotherapy versus the next day. Severity and duration of CIN were simultaneously captured by using AOC of ANC in this analysis, and potential covariates with imbalanced distributions were adjusted for when comparing AOC of ANC between the two treatment groups. Inclusion and exclusion criteria were applied in addition to the original criteria used in each trial (e.g., baseline ANC and body temperature and ANC measurement frequency within each chemotherapy cycle) to standardize patient selection in this analysis. Standardized definitions of study endpoints and covariates were also developed and applied in this study.

This study also has limitations. The original primary efficacy endpoint of the four clinical trials included in this analysis was duration of grade 4 neutropenia, and the trials were not designed to detect a difference in risk of infection/FN. The sample size in this pooled analysis was not sufficient to detect possible difference in the incidence proportions of FN between patients who received pegfilgrastim on the same day as chemotherapy versus the next day. Another limitation is that patients in the original studies did not have frequent enough measurements of ANC to allow examination of the ANC trajectory in all cycles of chemotherapy. In addition, patients enrolled in the original clinical trials might not be representative of patients with cancer treated in routine clinical practice today; thus, the results from this pooled analysis of clinical trial data might have limited generalizability.

## Conclusions

In this secondary analysis of individual patient data from four randomized clinical trials, we found that patients who received pegfilgrastim as indicated, on the day after chemotherapy, had less severe and less sustained suppression of ANC as manifested by higher ANC nadirs and smaller AOC of ANC than patients who received pegfilgrastim on the same day as chemotherapy. Next-day patients also had lower incidence proportions of grade 3 or 4 CIN than same-day patients. No significant differences were observed in the incidence proportions of FN, likely due to the lack of statistical power in the study. Together, these results support administration of pegfilgrastim as indicated, on the day after chemotherapy.

## Electronic supplementary material

Below is the link to the electronic supplementary material.

Supplementary material 1 (DOCX 60 kb)
