# Supplementary material for: Trajectory of absolute neutrophil counts in patients treated with pegfilgrastim on the day of chemotherapy versus the day after chemotherapy
Source: Cancer Chemother Pharmacol. 2016 Feb 17;77:703–12. doi: 10.1007/s00280-016-2970-5 (PMC4819939; doi:10.1007/s00280-016-2970-5)
Supplement: Supplementary file 1 — Supplementary material 1 (DOCX 60 kb) [file 280_2016_2970_MOESM1_ESM.docx]

# Online Resources

**Trajectory of absolute neutrophil counts in patients treated with pegfilgrastim on the day of chemotherapy versus the day after chemotherapy**

**Authors:** Yanli Li,^1^* Zandra Klippel,^2^ Xiaolong Shih,^3^ Hong Wang,^4^ Maureen Reiner,^5^ John H. Page^6^

**Affiliations:**

^1^Center for Observational Research, Amgen Inc., South San Francisco, CA

^2^Clinical Development, Amgen Inc., Thousand Oaks, CA

^3^SimulStat Inc., San Diego, CA

^4^TechData Service Company, LLC, King of Prussia, PA

^5^Global Biostatistical Science, Amgen Inc., Thousand Oaks, CA

^6^Center for Observational Research, Amgen Inc., Thousand Oaks, CA

***Corresponding Author:** Yanli Li, MD, PhD

Center for Observational Research

Amgen Inc.

1150 Veterans Blvd

South San Francisco, CA 94080

Phone: +1 (650) 244-2818

Email: [yanli.li@amgen.com](mailto:yanli.li@amgen.com)

Running head: ANC trajectory and timing of treatment with pegfilgrastim

Target journal: *Cancer Chemotherapy and Pharmacology*

## Online Resource 1 Selection of studies for comparison of same-day versus next-day pegfilgrastim administration

| **Selection Criteria** | **Number of Pegfilgrastim Trials** |
| --- | --- |
| Phase 2 or 3 clinical trials with individual patient data available | 28 |
| Pegfilgrastim indicated for chemotherapy-induced neutropenia | 24 |
| Conducted in adult patients with non-myeloid cancers | 23 |
| Had at least one ANC measurement at baseline of cycle 1 (days 1–4) and at least weekly measurements of ANC between day 4 and cycle end | 8 |
| Patients randomized to receive pegfilgrastim on the same day as chemotherapy or the next day | 4 |
| Had body temperature measured on a daily basis | 4 |
| Had evaluation of neutropenic infection within each chemotherapy cycle | 4 |

*ANC* absolute neutrophil count.

## Online Resource 2 Summary of studies

| **Study, Phase** | **Tumor Type** | **Primary Efficacy Endpoint** | **Chemotherapy Regimen** | **FN Risk Category** | **Publication** |
| --- | --- | --- | --- | --- | --- |
| Amgen study 20020134 (NCT00115193), phase 2 | Non-Hodgkin’s lymphoma | Duration of grade 4 neutropenia in cycle 1, defined as the number of days during cycle 1 for which ANC < 0.5 × 10^9^/L | R-CHOP (cyclophosphamide, doxorubicin, vincristine, prednisone, and rituximab) administered every 21 days for up to 6 cycles | Intermediate [[21](#_ENREF_21)] | Saven et al. 2006 [[30](#_ENREF_30)], Burris et al. 2010 [[29](#_ENREF_29)] |
| Amgen study 20020778 (NCT00115414), phase 2 | Breast cancer | Duration of grade 4 neutropenia in cycle 1, defined as the number of days during cycle 1 for which ANC (observed or imputed) was < 0.5 × 10^9^/L | TAC (docetaxel, doxorubicin, and cyclophosphamide) administered every 21 days for up to 6 cycles | High [[21](#_ENREF_21)] | Kaufman et al. 2004 [[37](#_ENREF_37)], Burris et al. 2010 [[29](#_ENREF_29)] |
| Amgen study 20030122 (NCT00113789), phase 2 | Relapsed or refractory ovarian cancer | Duration of grade 4 neutropenia in cycle 1, defined as the number of days during cycle 1 for which ANC < 0.5 × 10^9^/L | Topotecan 1.25 or 1.5 mg/m^2^/day administered days 1–5 every 21 days for up to 6 cycles | High [[21](#_ENREF_21)] | Burris et al. 2010 [[29](#_ENREF_29)] |
| Amgen study 20030123 (NCT00094822), phase 2 | Advanced or metastatic non-small cell lung cancer | Duration of grade 4 neutropenia in cycle 1, defined as the number of days during cycle 1 for which ANC < 0.5 × 10^9^/L | Carboplatin and docetaxel administered every 21 days for up to 6 cycles | Intermediate [[22](#_ENREF_22)] | Belani et al. 2006 [[31](#_ENREF_31)], Burris et al. 2010 [[29](#_ENREF_29)] |

*ANC* absolute neutrophil count; *FN* febrile neutropenia.

## Online Resource 3 Sensitivity analysis for time to ANC nadir for the studies in which chemotherapy was administered only on day 1 of the chemotherapy cycle

|  | **ANC Trajectory Metrics** | **Same Day** | **Next Day** | ***P* value^a^** |
| --- | --- | --- | --- | --- |
| Cycle 1 | Time to ANC nadir (days) |  |  |  |
|  | Mean ± SD (n^b^) | 7.46 ± 2.54 (90) | 7.50 ± 1.40 (90) |  |
|  | Median (Q1, Q3) | 7.00 (7.00, 8.00) | 7.00 (7.00, 8.00) | **0.020** |
|  | Time to ANC nadir distribution, n (%) |  |  |  |
|  | < 7 days | 21 (23.3) | 7 (7.8) | **0.030** |
|  | 7 days | 40 (44.4) | 43 (47.8) |  |
|  | 8 days | 21 (23.3) | 30 (33.3) |  |
|  | > 8 days | 8 (8.9) | 10 (11.1) |  |
| Cycle 3^c^ | Time to ANC nadir (days) |  |  |  |
|  | Mean ± SD (n^a^) | 7.41 ± 0.64 (27) | 7.45 ± 0.57 (29) |  |
|  | Median (Q1, Q3) | 7.00 (7.00, 8.00) | 7.00 (7.00, 8.00) | 0.650 |

Analyses in cycle 1 include data from eligible patients in Amgen studies 20020134, 20020778, and 20030123 and in cycle 3 include data from eligible patients in Amgen studies 20020778.

^a^ Wilcoxon rank sum test was used to test differences of time to nadir (continuous), and chi-square test was used to test difference of time to nadir distribution (< 7 days, 7 days, 8 days, and > 8 days) between same-day versus next-day patients.

^b^ The n for each parameter (of each treatment arm) is the number of patients eligible for the corresponding statistics in cycle 1 or cycle 3.

^c^ Analysis for difference of time to nadir distribution (< 7 days, 7 days, 8 days, and > 8 days) between same-day versus next-day patients was not performed for cycle 3, due to small sample size.

*ANC* absolute neutrophil count; *Q1* quartile 1; *Q3* quartile 3; *SD* standard deviation.

## Online Resource 4 Sensitivity analysis for time to ANC nadir for the study in which chemotherapy was administered over the first 5 days of the chemotherapy cycle

|  | **ANC Trajectory Metrics** | **Same Day** | **Next Day** | ***P* value^a^** |
| --- | --- | --- | --- | --- |
| Cycle 1 | Time to ANC nadir (days) |  |  |  |
|  | Mean ± SD (n^b^) | 8.60 ± 0.55 (5) | 9.33 ± 0.52 (6) |  |
|  | Median (Q1, Q3) | 9.00 (8.00, 9.00) | 9.00 (9.00, 10.00) | 0.071 |
| Cycle 3 | Time to ANC nadir (days) |  |  |  |
|  | Mean ± SD (n^a^) | 8.67 ± 0.58 (3) | 9.50 ± 0.58 (4) |  |
|  | Median (Q1, Q3) | 9.00 (8.00, 9.00) | 9.50 (9.00, 10.00) | 0.168 |

Analyses in cycles 1 and 3 include data from eligible patients in Amgen study 20030122.

^a^ Wilcoxon rank sum test was used to test differences of time to nadir (continuous) between same-day versus next-day patients.

^b^ The n for each parameter (of each treatment arm) is the number of patients eligible for the corresponding statistics in cycle 1 or cycle 3.

*ANC* absolute neutrophil count; *Q1* quartile 1; *Q3* quartile 3; *SD* standard deviation.
